# Population pharmacokinetics and CSF penetration of flucytosine in adults with HIV-associated cryptococcal meningoencephalitis

**DOI:** 10.1093/jac/dkad038

**Published:** 2023-03-01

**Authors:** Katharine E Stott, Ajisa Ahmadu, Cheusisime Kajanga, Melanie Moyo, Ebbie Gondwe, Wezzie Chimang’anga, Madalitso Chasweka, Jennifer Unsworth, Ana Jimenez-Valverde, Bhavana Jagota, Reya V Shah, David S Lawrence, David G Lalloo, Tom Harrison, Joseph N Jarvis, William Hope, Henry C Mwandumba

**Affiliations:** Antimicrobial Pharmacodynamics and Therapeutics, Department of Molecular and Clinical Pharmacology, University of Liverpool, Liverpool, UK; Malawi Liverpool Wellcome Trust Clinical Research Programme, Blantyre, Malawi; Malawi Liverpool Wellcome Trust Clinical Research Programme, Blantyre, Malawi; Malawi Liverpool Wellcome Trust Clinical Research Programme, Blantyre, Malawi; Malawi Liverpool Wellcome Trust Clinical Research Programme, Blantyre, Malawi; Department of Medicine, Kamuzu University of Health Sciences, Blantyre, Malawi; Malawi Liverpool Wellcome Trust Clinical Research Programme, Blantyre, Malawi; Malawi Liverpool Wellcome Trust Clinical Research Programme, Blantyre, Malawi; Malawi Liverpool Wellcome Trust Clinical Research Programme, Blantyre, Malawi; Antimicrobial Pharmacodynamics and Therapeutics, Department of Molecular and Clinical Pharmacology, University of Liverpool, Liverpool, UK; Antimicrobial Pharmacodynamics and Therapeutics, Department of Molecular and Clinical Pharmacology, University of Liverpool, Liverpool, UK; Antimicrobial Pharmacodynamics and Therapeutics, Department of Molecular and Clinical Pharmacology, University of Liverpool, Liverpool, UK; Institute for Infection and Immunity, St George’s University London, London, UK; Department of Clinical Research, Faculty of Infectious and Tropical Diseases, London School of Hygiene and Tropical Medicine, London, UK; Botswana Harvard AIDS Institute Partnership, Gaborone, Botswana; Liverpool School of Tropical Medicine, Liverpool, UK; Clinical Academic Group in Infection, St George’s University Hospitals NHS Foundation Trust, London, UK; MRC Centre for Medical Mycology, University of Exeter, Exeter, UK; Department of Clinical Sciences, Liverpool School of Tropical Medicine, Liverpool, UK; Department of Clinical Research, Faculty of Infectious and Tropical Diseases, London School of Hygiene and Tropical Medicine, London, UK; Botswana Harvard AIDS Institute Partnership, Gaborone, Botswana; Antimicrobial Pharmacodynamics and Therapeutics, Department of Molecular and Clinical Pharmacology, University of Liverpool, Liverpool, UK; Malawi Liverpool Wellcome Trust Clinical Research Programme, Blantyre, Malawi; Department of Medicine, Kamuzu University of Health Sciences, Blantyre, Malawi; Department of Clinical Sciences, Liverpool School of Tropical Medicine, Liverpool, UK

## Abstract

**Background:**

There are limited data describing clinical flucytosine pharmacokinetics (PK). The variability of flucytosine partitioning into the CNS is not known. We described the interindividual variability in flucytosine PK in patients with HIV-associated cryptococcal meningoencephalitis. In addition, we quantified the extent and variability of CSF partitioning of flucytosine.

**Methods:**

A PK study was conducted in 64 patients with confirmed HIV-associated cryptococcal meningoencephalitis in Blantyre, Malawi. A four-compartment PK model was developed, and Monte Carlo simulations were performed with flucytosine administered at different doses and in different schedules.

**Results:**

The estimated mean apparent volume of the central compartment was 17.50 (SD 9.99) L; mean apparent clearance was 5.88 (SD 3.35) L/h; mean apparent volume of the CNS compartment was 41.73 (SD 13.66) L. From the Bayesian posterior estimates, AUC_24_ values at steady state (144–168 h) with doses of 25 mg/kg q6h were median (IQR) 890.38 (603.81–1213.70) mg.h/L in plasma and 595.66 (425.69–776.64) mg.h/L in CSF. The ratio of CSF:plasma AUC_24_ was 0.69 (IQR 0.58–0.82).

**Conclusions:**

This study revealed significant interindividual variability in flucytosine PK in plasma and CSF in patients with HIV-associated cryptococcal meningoencephalitis. The population PK model is a first critical step for revised flucytosine regimens that maximize fungal killing and minimize toxicity and the emergence of resistance.

## Introduction

Flucytosine was first developed in 1957 within antitumour drug discovery programmes.^1^ In the decade that followed its antifungal properties were recognized, and by 1968 flucytosine was used in humans for the treatment of candidiasis and cryptococcosis.^2,3^ Flucytosine is a pyrimidine analogue that lacks intrinsic antifungal activity, but is taken up by susceptible fungal cells and deaminated intracellularly to its active metabolite, 5-fluorouracil, by fungal cytosine deaminase.^4^ The further conversion of 5-fluorouracil produces metabolites that inhibit fungal RNA and DNA synthesis.^5^ Following oral administration, flucytosine exhibits high oral bioavailability (approximately 90%).^6,7^ The success of flucytosine in the treatment of cryptococcal meningoencephalitis is in part due to its excellent CNS partitioning. Flucytosine reaches high concentrations in brain parenchyma and CSF, with CSF:plasma ratios of 0.74–0.84 reported from clinical samples within 2 h of dosing.^8–10^ Physicochemical attributes of flucytosine that favour good CNS penetration include its negligible protein binding, low molecular weight and polarity.^11^

Despite over half a century of experience with the use of flucytosine to treat cryptococcal meningitis, there remains uncertainty surrounding the optimal dosing strategy for antifungal effect and to minimize the risk of flucytosine resistance. The aim of this study was to describe the interindividual variability in flucytosine exposure in plasma and CSF in patients with HIV-associated cryptococcal meningoencephalitis. In addition, we sought to quantify the extent and variability of the CSF penetration of flucytosine in this cohort. We interrogated several clinical covariates for their impact on flucytosine pharmacokinetics (PK). We then employed Monte Carlo simulation to suggest the impact of modified dosing strategies on flucytosine exposure.

## Materials and methods

### Clinical PK study

The PK data were collected within a substudy of the Phase III AMBIsome Therapy Induction OptimisatioN (AMBITION-cm) trial for HIV-associated cryptococcal meningitis.^12^ Patients with HIV-associated cryptococcal meningoencephalitis were randomized to either a single high dose of liposomal amphotericin B (LAmB) (AmBisome, Gilead Sciences Inc.) 10 mg/kg/day in combination with 14 days of flucytosine 100 mg/kg/day plus fluconazole 1200 mg/day (intervention arm), or 7 days of amphotericin B deoxycholate (DAmB) 1 mg/kg/day plus flucytosine 100 mg/kg/day, followed by 7 days of fluconazole 1200 mg/day (control arm).^12^ PK data were collected from patients recruited to AMBITION-cm at Queen Elizabeth Central Hospital in Blantyre, Malawi.

Flucytosine was administered orally, or via nasogastric tube, in four divided doses of 25 mg/kg spaced 6 h apart. In the event of deterioration in renal function (reduction in creatinine clearance, CrCl), the dosing interval was increased: participants with CrCl 20–40 mL/min received 25 mg/kg flucytosine q12h; those with CrCl 10–20 mL/min received 25 mg/kg flucytosine q24h; those with CrCl <10 mL/min were dosed less frequently than q24h, at the discretion of the local trial principal investigator. Administration times were documented in real time by patients, caregivers or nursing staff. Blood samples were collected on day 1 at 0, 2, 4, 7, 12 and 23 h after the first dose of flucytosine, and then on day 7 at the same times. A volume of 2 mL of blood was collected into heparinized collection tubes and placed on ice at the bedside. Lumbar punctures were performed according to the AMBITION-cm study protocol on days 1, 7 and 14 and more frequently if required to manage raised intracranial pressure. Within 30 min of collection, samples were centrifuged at 1500 g for 10 min at 4°C. Plasma and CSF supernatant was stored at −80°C until shipment to the University of Liverpool, UK.

### Ethics

Ethical approval for the PK substudy of AMBITION-cm was granted by the Research Ethics Committee of the London School of Hygiene and Tropical Medicine (14355) and by the Malawi National Health Sciences Research Committee (1907). All patients who had capacity to do so provided written informed consent for participation in the trial and then separately for inclusion in the PK substudy. Where patients were incapacitated, consent was obtained from a next of kin with legal responsibility and then reattempted with the patient if appropriate in accordance with their clinical status.

### Measurement of flucytosine concentrations

Flucytosine was extracted from human plasma and CSF as follows. The internal standard, [2H 15N]5FC (Alsachim, France), was prepared in acetonitrile (2.5 mg/L, Fisher Scientific UK) and 100 μL was added to a 96-well protein precipitation plate (Phenomenex, Cheshire, UK). Then, 25 μL of each of patient samples, blanks, calibrators (range 0.1–100 mg/L) and quality controls (0.1, 0.75, 7.5 and 75 mg/L) was mixed with the internal standard on an orbital shaker for 5 min at 400 rpm. Liquid was drawn through the protein precipitation plate using a positive-pressure manifold. Water and 0.1% formic acid (1000 µL) were added to each well before mixing on an orbital shaker for 5 min at 400 rpm.

LC-MS-MS was carried out using a Waters Acquity UPLC system coupled to a Waters Xevo TQ-XS triple quadrupole mass spectrometer fitted with an electrospray source. The LC-MS system was controlled using MassLynx Security software (Version 4.2). Analytes were injected (2 µL) onto a Waters HSS T3 column (2.1 mm × 100 mm, 1.8 µm, 40°C) and separated over a 3.5 min gradient using a mixture of solvents A and B. Solvent A was LC-MS-grade water with 0.1% (v/v) formic acid. Solvent B was HPLC-grade acetonitrile with 0.1% (v/v) formic acid. Separations were performed by applying a linear gradient of 5% to 95% solvent B over 2 min at 0.4 mL/min followed by an equilibration step (1.5 min at 5% solvent B).

Prior to sample analysis, the analytical method was validated to assess selectivity, recovery and matrix effects, interday and intraday accuracy and precision, carryover, dilution integrity, stability in the matrix (4 h at room temperature, three freeze-thaw cycles and 5 days at 4°C), and processed sample stability (reinjection of extracts after 24 h). The lower limit of quantitation (LLQ) was defined as 0.1 mg/L. The interday and intraday coefficient of variation (CV%) on the four quality control levels ranged from 5.82% to 8.58% and 6.01% to 7.01%, respectively.

### Population PK modelling

PK data were analysed using the non-parametric adaptive grid algorithm of the program Pmetrics version 2.0.0 for R version 4.2.0.^13^ Various four-compartment structural models were explored to fit patient data, with bolus input into the absorptive compartment (i.e. gut) and first-order elimination kinetics from the central compartment. First-order intercompartmental rate constants were used. Both mean and median parameter values were examined. For each model, additive and multiplicative error models were assessed and optimized. Different approaches for handling observed flucytosine concentrations below the LLQ were tested: those observations were set to zero in one tested dataset, and to half the LLQ (0.05 mg/L) in an alternative tested dataset. The fit of the various models to the data was assessed and compared based on diagnostic plots, including observed versus predicted concentrations before and after the Bayesian step, residuals versus predicted concentrations, and visual predictive plots. The Akaike information criterion, log-likelihood value, mean weighted error (a measure of bias), and bias-adjusted mean weighted squared error (a measure of precision) were also assessed and used to compare models.

### Population PK covariate screening

Bidirectional stepwise multivariate linear regression was conducted to assess for associations between clinical covariates and LAmB PK. Patient age, weight, sex, baseline alanine aminotransferase (ALT) level, baseline serum creatinine level and baseline CrCl (by Cockroft–Gault equation) were investigated as independent predictors of the Bayesian posterior estimates of PK parameters from the baseline model.

### Monte Carlo simulations

Monte Carlo simulations (*n* = 5000 per simulated regimen) were performed in Pmetrics^13^ and confirmed using ADAPT 5.^14^ In Pmetrics, the non-parametric support points from the population PK model (i.e. a vector of one value for each of the model parameters and the associated probability of that set of parameter values) served as the mean of one multivariate normal distribution in a joint distribution. Each multivariate distribution was weighted by the probability of the associated support point. Simulations were performed with a simulated weight distribution that took its mean, SD and limits from the respective values in the patient population. In ADAPT, values for the system parameters were randomly selected from a log normal distribution, with mean and the diagonal of the covariance matrix derived from the original model fit in Pmetrics.

For both sets of simulations, flucytosine was administered in three different fractionation schedules. Overall dosages of 100 mg/kg/day were administered as 25 mg/kg q6h, 50 mg/kg q12h and 100 mg/kg q24h. Simulated AUC values in plasma and CSF from 144 to 168 h (at steady state) were calculated in Pmetrics using trapezoidal approximation, with prediction intervals of 0.1 h.

### Toxicity

Potential relationships between drug exposure and toxicity were explored. The Bayesian posterior PK predictions from the population model were used to estimate AUC_144–168_, AUC_0–168_ and the maximum concentration in the dosing window (*C*_max_). Toxicity was defined according to the Division of AIDS Table for Grading the Severity of Adult and Paediatric Adverse Events, version 2.1,^15^ as any of the following grade 3 or 4 adverse events occurring after the start of flucytosine therapy: haemoglobin ≤9.0 g/dL in males or ≤8.5 g/dL in females; WBC <1.5 × 10^9^ cells/L; neutrophils <0.6 × 10^9^ cells/L; platelets <50 × 10^9^ cells/L; ALT ≥180 IU/L. Logistic regression was used to explore the relationship between estimated AUC_144–168_ and AUC_0–168_ and the development of these parameters.

## Results

### Patients

In total, 64 patients were recruited over an 11-month period between November 2018 and October 2019. Thirty-one of those patients were in the control arm and 33 in the single-dose intervention arm. All 64 patients had cryptococcal meningoencephalitis confirmed by a positive India ink stain or cryptococcal antigen test (CrAg lateral flow assay, IMMY) from a CSF sample. Twenty-four patients (37%) were female. Patient characteristics expressed as median (IQR) were as follows: age 36 years (33–41 years); weight 50 kg (47–56 kg); CrCl at enrolment 105.15 mL/min (80.63–123.05 mL/min). This was a cohort characterized by advanced immunosuppression: the median CD4 T-cell count was 39 cells/mm^3^ (IQR 21–83 cells/mm^3^, *n* = 57).

### PK data

The dataset included 595 plasma flucytosine observations and 209 CSF flucytosine observations, with an arithmetic mean of 9.2 plasma PK samples per patient and 3.5 CSF PK samples per patient. In total, 34 plasma observations and 82 CSF observations were below the LLQ. Of 64 patients, 7 (11%) missed at least one dose of flucytosine. In two cases, this was due to the development of thrombocytopenia. In three cases, the patient’s clinical condition prevented perfect medication adherence: for example, low conscious level with no feeding tube in situ. In two cases, medication administration was unsupervised, and patients did not take all doses as prescribed.

### Population PK analysis

The final model comprised an absorption compartment, central compartment, peripheral compartment and CSF compartment. Mean PK parameter estimates fitted the data better than median estimates and were used for Bayesian estimates of drug exposure for individual patients. A better model fit was attained when flucytosine concentrations below the LLQ were set to 0.05 mg/L than when those observations were set to zero. The chosen base model took the form:


(1) $$\frac{dX(1)}{dt}=-Ka\;*\;X(1)$$

(2) $$\begin{array}{rl} \frac{dX(2)}{dt}\;=\; & Ka\;*\;X(1)-\left(K23+K24+\left(\frac{CL}{V}\right)\;*\;X(2)\right)\\ & +(K32\;*\;X(3))+(K42\;*\;X(4)) \end{array}$$

(3) $$\frac{dX(3)}{dt}=K23\;*\;X(2)-K32\;*\;X(3)$$

(4) $$\frac{dX(4)}{dt}=K24\;*\;X(2)-K42\;*\;X(4)$$

Equations 1 to 4 describe the rate of change in the amount of flucytosine in the gastrointestinal tract, circulation, CNS and peripheral compartment, respectively. *K*a is the absorption rate constant from the gut to the central compartment. *X*(1), *X*(2), *X*(3) and *X*(4) are the amounts of flucytosine in the respective compartments, in milligrams. *K*23 and *K*32 are the first-order transfer constants connecting the central compartment and the CNS compartment. *K*24 and *K*42 are the first-order transfer constants connecting the central compartment and the peripheral compartment. *CL* is the first-order clearance of drug from the central compartment in litres per hour. The volume of the central compartment is represented by *V*. Output equations took the form:


(5) Y(1)=X(2)/V

(6) Y(2)=X(3)/Vcns

In the output equations, *Y*(1) and *Y*(2) are the concentrations of drug in the central and CNS compartments, respectively. Vcns is the volume of the CNS compartment.

Multivariate linear regression of each subject’s clinical covariates against the Bayesian posterior parameter values revealed no significant correlations. Specifically, patient weight did not significantly correlate with volume of distribution, and neither plasma creatinine level nor CrCl significantly correlated with flucytosine clearance. Because there are consistent data relating flucytosine clearance to CrCl,^16–18^ the model was nevertheless run with *CL* scaled to CrCl. However, no improvement in model fit could be demonstrated and consequently the base model was chosen as the final model.

**Figure 1.**
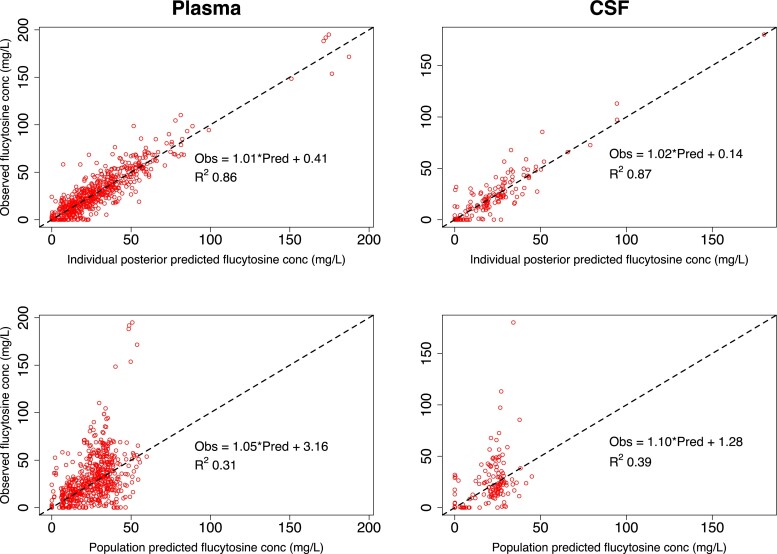
Scatterplots of observed versus predicted values in plasma and CSF, for the chosen population PK model. Goodness-of-fit plots of observed versus mean predicted flucytosine concentrations for the chosen population PK model after the Bayesian step. Upper plots show the fit of the mean individual-level posterior PK estimates in plasma and CSF. Lower plots show the fit of the population-level PK estimates. Circles represent observed-predicted data points. Dashed lines represent the line of identity. conc, concentration; Obs, observed flucytosine concentration; Pred, predicted flucytosine concentration. This figure appears in colour in the online version of *JAC* and in black and white in the print version of *JAC*.

**Figure 2.**
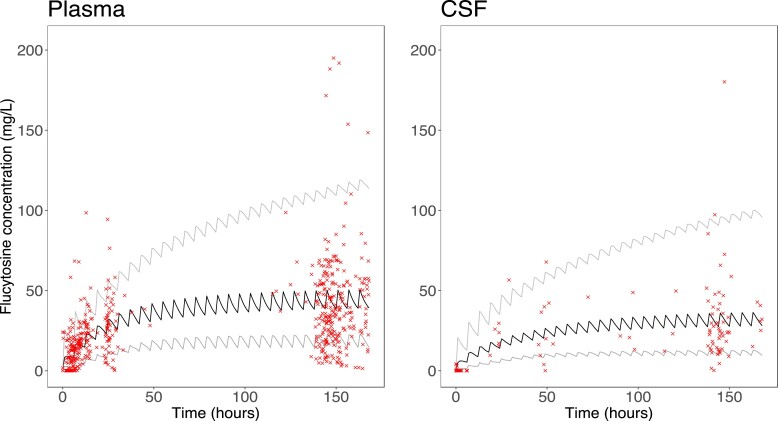
Visual predictive check. Simulations were performed to predict flucytosine concentrations in patients administered the standard regimen of 25 mg/kg q6h. Median (black line), 5th percentile and 95th percentile (grey lines) flucytosine concentrations were plotted per hour over 168 h. Observed flucytosine concentrations from our cohort were overlayed (crosses). Overall, 76% of plasma concentrations and 86% of CSF concentrations fell within the predicted 5th and 95th percentiles. This figure appears in colour in the online version of *JAC* and in black and white in the print version of *JAC*.

The population PK parameter estimates from the final model are displayed in Table 1. Mean estimates provided a slightly better model fit than did median estimates. Terminal half-life in plasma was approximately 14.5 h. Linear regression models of the individual and population observed versus predicted values in plasma and CSF are displayed in Figure 1. Bias in the population fits was low: −0.11 in plasma and −0.06 in CSF. A visual predictive check revealed that 76% of observed plasma concentrations and 86% of observed CSF concentrations of flucytosine fell within the 5th and 95th percentiles of concentrations predicted by the final model (Figure 2). The model did not reliably predict flucytosine concentrations of zero or close to zero. This likely reflects the fact that the simulations did not account for inaccuracies in medication administration or documentation that may have arisen in the clinical cohort.

**Table 1.** Flucytosine pharmacokinetic model parameter estimates

Parameter	Mean	SD	Median	CV%
*K*a (h^−1^)	1.77	1.81	1.01	102.06
CL/F (L/h)	5.88	3.35	5.31	56.93
V/F (L)	17.50	9.99	14.76	57.06
*K*23 (h^−1^)	15.55	4.97	18.57	31.97
*K*32 (h^−1^)	9.02	5.43	6.69	60.21
*K*24 (h^−1^)	5.68	7.60	1.09	133.77
*K*42 (h^−1^)	1.38	2.33	0.24	168.39
Vcns/F (L)	41.73	13.66	44.55	32.74

*K*a, absorption rate constant from gut to central compartment; CL/F, apparent clearance; *K*23, first-order transfer constant from central compartment to CNS compartment; *K*32, first-order transfer constant from CNS compartment to central compartment; *K*24, first-order transfer constant from central compartment to peripheral compartment; *K*42, first-order transfer constant from peripheral compartment to central compartment; Vcns/F, apparent volume of CNS compartment; V/F, apparent volume of central compartment; CV%, coefficient of variation.

### Flucytosine penetration into CSF

The AUC values generated from each patient’s posterior PK estimates were highly variable. The median (IQR) plasma AUC_144–168_ following 100 mg/kg/day flucytosine in four split doses (q6h) was 890.38 (603.81–1213.70) mg.h/L. The median (IQR) CSF AUC_144–168_ was 595.66 (425.69–776.64) mg.h/L. The time interval of 144 to 168 h was chosen in order to estimate flucytosine exposure at steady state. From these posterior estimates, the median ratio of CSF AUC_144–168_/plasma AUC_144–168_ was 0.69 (IQR 0.58–0.82).

### Simulations

**Figure 3.**
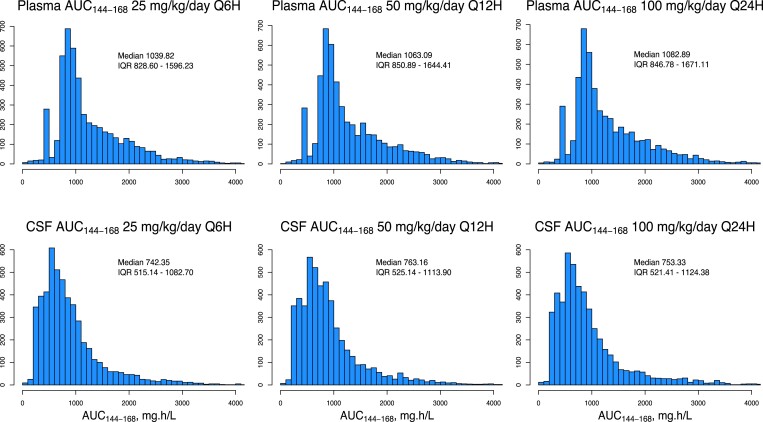
Simulated AUC_144–168_ in plasma and CSF resulting from various flucytosine dosing regimens, based on the non-parametric prior distribution from the final PK model. This figure appears in colour in the online version of *JAC* and in black and white in the print version of *JAC*.

Monte Carlo simulations were employed to investigate the distribution of AUC_144–168_ for overall dosages of flucytosine of 100 mg/kg/day, in fractionation schedules of q6h, q12h and q24h. The AUC distributions resulting from these simulations were almost identical in each fractionation schedule, as would be expected with linear PK (Figure 3). Summary statistics of flucytosine exposure at steady state in the 5000 simulated profiles administered 25 mg/kg q6h are displayed in Table 2.

**Table 2.** Summary statistics of flucytosine exposure in plasma and CSF for 5000 simulated patients administered flucytosine 25 mg/kg q6h

	25th percentile	Median	75th percentile
Plasma			
ȃ*C*_min_ (mg/L)	26.8	38.0	63.5
ȃ*C*_max_ (mg/L)	44.9	50.6	74.0
CSF			
ȃ*C*_min_ (mg/L)	15.3	23.9	43.5
ȃ*C*_max_ (mg/L)	28.1	41.9	49.5

Simulated PK profiles (*n* = 5000) administered flucytosine 25 mg/kg q6h were ordered by plasma AUC_144–168_ and then by CSF AUC_144–168_ to identify the profile on the median, 25th and 75th percentiles of flucytosine exposure at steady state. The *C*_min_ and *C*_max_ values from the respective profiles are displayed. *C*_min_, minimum concentration in the dosing window; *C*_max_, maximum concentration in the dosing window.

### Toxicity

Anaemia was present prior to the start of treatment in 22% of patients (*n* = 15). In addition, 48% of patients (*n* = 31) either developed a new grade 3 or 4 anaemia or moved from grade 3 to grade 4 anaemia during treatment with flucytosine. One patient had a WBC count <1.5 × 10^9^/L before treatment; 11% of patients (*n* = 7) had evidence of grade 3 to 4 reduction in WBC after treatment had started. Whereas no patients were neutropenic before starting treatment, 14% of patients (*n* = 9) developed grade 3 to 4 neutropenia on treatment. One patient had thrombocytopenia prior to the start of treatment, and 8% of patients (*n* = 5) developed a grade 3 to 4 thrombocytopenia on treatment. No patients had a raised ALT at enrolment and one patient had grade 3 to 4 rise in ALT after treatment had started. Four of the 64 patients (6%) were predicted (from their Bayesian posterior estimates) to mount a *C*_max_ in the range associated with toxicity (>100 mg/mL).^17^ No clinically significant associations were identified between flucytosine AUC_144–168_, AUC_0–168_ or *C*_max_ and the development of these markers of toxicity.

## Discussion

To our knowledge, this is the first study of flucytosine population PK in cryptococcal meningoencephalitis patients. We modelled a uniquely comprehensive clinical dataset and estimated the PK of flucytosine at the population level, its penetration into the CNS and the variability surrounding those estimates. The reason that no relationship was apparent between CrCl and flucytosine clearance may be due to the relatively narrow range of CrCl in this patient population.

Substantial variability was apparent in simulated AUC values in both plasma and CSF, as well as in the degree of flucytosine partitioning from plasma to CSF. The latter finding is consistent with previous studies of antimicrobial dose optimization in the setting of variable meningeal inflammation.^19–21^ A detailed understanding of flucytosine population PK is critical for rational prescribing and will support several ongoing efforts to improve the treatment of cryptococcal meningoencephalitis. These include the refinement of combination induction therapy, the development of sustained-release formulations of flucytosine and the exploration of reduced overall doses of flucytosine, with associated reduction in the risk of toxicity.

Flucytosine is an essential component of effective combination induction therapy for cryptococcal meningoencephalitis. The coadministration of flucytosine (100 mg/kg/day) with fluconazole (1200 mg/day) suppresses the emergence of resistant subpopulations that otherwise develop on fluconazole monotherapy^22,23^ and is associated with mortality outcomes that are comparable to DAmB-based induction regimens.^24^ As a partner drug with DAmB, flucytosine produces faster fungicidal activity than fluconazole does.^24,25^ In order to optimize the use of flucytosine in combination treatment regimens for cryptococcal meningoencephalitis, an understanding of its PK at the population level is required. For example, it may be the case that the optimal dosing strategy of flucytosine varies depending on the drug with which it is coadministered. In the current era of increasing antifungal resistance,^26^ it is possible that regional differences in susceptibility patterns will warrant different prescribing strategies. The present analysis provides novel insight into the population PK of flucytosine to support efforts to address these questions.

Combination therapy strategies for cryptococcal meningoencephalitis have been arrived at through painstaking and time-consuming manipulation of treatment regimens using the same limited number of drugs over a period of 40 years.^27^ This approach has been necessary but it is inefficient. We are now in a position to modernize the approach to designing treatment regimens for cryptococcal meningoencephalitis; we have a validated pharmacodynamic (PD) surrogate of all-cause mortality (early fungicidal activity; EFA)^28^ and a host of preclinical methods and pharmacometric modelling techniques that enable investigation and simulation of novel regimens in an environment free of clinical risk.^29,30^ The success of adaptive trial designs in assessing potential treatment regimens for COVID-19 such as AGILE (ISRCTN27106947) and RECOVERY (ISRCTN50189673) has highlighted the speed and efficiency with which novel regimens can undergo clinical evaluation. The detailed estimates of flucytosine PK provided by the current analysis can support a rational approach to the design of studies that include flucytosine as induction therapy.

The flucytosine PD index that correlates most closely with efficacy in *Candida* is the fraction of time that the concentration of drug is above the MIC within the dosing window: %T > MIC.^31,32^ Toxicity is associated with peak plasma concentrations >100 mg/L.^17^ Flucytosine is therefore administered as a split dose, four times per day to maximize %T > MIC while minimizing the potential for toxic *C*_max_ levels. This frequent dosing is inconvenient and has been cited as a barrier to routine flucytosine use in low-income settings where staff-to-patient ratios are low.^33^ A Phase I trial was recently launched by the 5FC HIV-Crypto consortium to study a sustained-release formulation of flucytosine that could be administered twice daily.^34,35^ Importantly, the magnitude of %T > MIC that best correlates with antifungal activity of flucytosine in cryptococcal meningoencephalitis has not been defined; neither has the target that prevents the development of flucytosine resistance. We were therefore unable to perform a probability of PD target attainment analysis for flucytosine in cryptococcal meningoencephalitis. Although our simulations demonstrate that flucytosine AUC at steady state is comparable per overall daily dose regardless of fractionation schedule, this is not to suggest that %T > MIC will be comparable across different dosing schedules. Studies to define the clinical PD of flucytosine are not likely to be forthcoming given that combination antifungal therapy is a standard of care and flucytosine monotherapy is associated with the rapid development of resistance.^4,5^ Preclinical studies to address these data gaps are warranted, as have been conducted in *Candida*.^31,32^

There are signals that it may be possible to reduce the dose of flucytosine from 100 mg/kg/day without compromising antifungal efficacy. For example, in combination with LAmB, a murine bridging study suggested that a lower flucytosine dosage of 50 mg/kg/day may be appropriate and associated with antifungal effect comparable to 100 mg/kg/day.^10^ In Thai patients with advanced HIV disease given oral flucytosine 100 mg/kg/day, plasma and CSF levels were significantly lower than levels achieved following intravenous dosing. Despite this, EFA was not significantly different between the two patient groups, when flucytosine was given in combination with DAmB.^8^ Of note, our analysis does not support overall dosage reduction from 100 mg/kg/day, because the nature and magnitude of the PK/PD target has not been defined for flucytosine in cryptococcal meningoencephalitis. If dose reduction were possible without reduction in efficacy, this could go some way to improving the affordability of flucytosine, and may reduce the need for frequent monitoring for toxicity. Again, any reduction in flucytosine levels would require consideration of the achievement of sufficient exposure not only for efficacy but also to prevent the emergence of resistant subpopulations. The present analysis adds valuable insight to support any such redesign of flucytosine regimens, although further preclinical experiments are required.

A limitation of this study is that flucytosine concentrations below the LLQ were set to a uniform value of 0.05 mg/L, which likely contributed to the inability of the final model to predict the lowest flucytosine concentrations. This approach was chosen because it led to overall better model fit than the alternative strategy for handling these datapoints by setting them to 0 mg/L. A higher sensitivity bioanalytical assay may have improved model accuracy in this regard.

In conclusion, this study has revealed significant interindividual variability in flucytosine PK in plasma and CSF in patients with HIV-associated cryptococcal meningoencephalitis. The population parameters estimated from the final model provide the PK foundation for further optimization of flucytosine dosing for this neglected and frequently lethal infection.

## Supplementary Material

dkad038_Supplementary_Data

## Transparency declarations

TH and JNJ were recipients of the investigator award (to institution) for the Phase II study, and have received speaker fees from Gilead Sciences. WH holds or has recently held research grants with F2G, Astellas Pharma, Spero Therapeutics, Antabio, Allecra, Bugworks and NAEJA-RGM. He holds awards from the Medical Research Council, National Institutes of Health Research, the FDA and the European Commission. WH has received personal fees in his capacity as a consultant for F2G, Amplyx, Ausperix, Spero Therapeutics, VenatoRx, Pfizer and BLC/TAZ. All other authors have no transparency declarations to declare.
