# Supplementary material for: Population pharmacokinetics and CSF penetration of flucytosine in adults with HIV-associated cryptococcal meningoencephalitis
Source: J Antimicrob Chemother. 2023 Mar 1;78(4):1015–22. doi: 10.1093/jac/dkad038 (PMC10068416; doi:10.1093/jac/dkad038)
Supplement: dkad038_Supplementary_Data [file dkad038_supplementary_data.docx]

Supplementary data

**Model file used in Pmetrics for the final covariate model for flucytosine**

#Primary variables

Ka, 0.10, 7.00

V, 0.10, 40.00

K23, 0.01, 20.00

K32, 0.01, 20.00

K24, 0.01, 20.00

K42, 0.01, 20.00

Vcns, 0.10, 60.00

SCL, 0.01, 15.00

#F (bioavailability term; used here to model dose according to patient weight)

FA(1)=WT

#Differential equations

XP(1)= -Ka*X(1)

XP(2)= -(Kcp + Kcs + (SCL/V))*X(2) + (Ksc*X(3)) + (Kpc*X(4)) + (Ka*X(1))

XP(3)= Kcs*X(2) - Ksc*X(3)

XP(4)= Kcp*X(2) - Kpc*X(4)

#Covariates

WT

#Output equations

Y(1)=X(2)/V

Y(2)=X(3)/Vcns

#Error model

L=3

0.025,0.100,0.000,0.000

0.025,0.100,0.000,0.000

Ka, absorption rate constant from gut to central compartment; V, flucytosine volume of distribution, central compartment; K23, first-order transfer constant from central compartment to CNS compartment; K32, first-order transfer constant from CNS compartment to central compartment; K24, first-order transfer constant from central compartment to peripheral compartment; K42, first-order transfer constant from peripheral compartment to central compartment; Vcns, flucytosine volume of distribution, CNS compartment, SCL, clearance of flucytosine from central compartment.
